# Evidence-based healthcare for all: a new era?

**DOI:** 10.1590/S1516-31802008000100001

**Published:** 2008-01-03

Is it possible to hold a major medical congress that provides diagnostic and therapeutic updates, independent of for-profit companies? The results from the Fifteenth Cochrane Colloquium (Evidence-Based Healthcare for All, in São Paulo, October 23 to 28, 2007) have demonstrated that the answer to this question is a resounding YES!

The Congress was held entirely with funds from the participants and from public-interest bodies such as the Associação Paulista de Medicina (APM), the Ministry of Health (Department of Science and Technology and Department of Education and Management), the National Agency for Sanitary Surveillance (Agência Nacional de Vigilância Sanitária, Anvisa), the National Health Agency (Agência Nacional de Saúde, ANS), the State of São Paulo Federation of Unimed Healthcare Cooperatives (Federação Estadual das Unimeds de São Paulo), the Regional Library of Medicine (Biblioteca Regional de Medicina, Bireme) [Pan-American Health Organization/World Health Organization, PAHO/WHO] and the State of São Paulo Regional Medical Council (Conselho Regional de Medicina do Estado de São Paulo, Cremesp).

The Congress satisfied all types of participants from Brazil and various types of professionals attended, including judges and prosecuting attorneys with an interest in Evidence-Based Medicine. There were also foreign participants from around 50 countries. It was held in a large hotel and its opening included a high-level show of Brazilian Popular Music (MPB) featuring Mônica Salmaso, Teco Cardoso and Toninho Ferragutti. There were also soccer matches in which a team composed of members of the national team of 1982 and other World Cups played against teams of congress participants (including a women's team), tropical dance classes and a dinner dance. These counterbalanced intense scientific activities that included one of the most complete courses on evidence-based technological evaluations that had ever taken place in Brazil or elsewhere.

Many colleagues from abroad considered the Congress to be the best in the history of the Cochrane Collaboration! Thus, it was possible to show to ourselves and to our foreign visitors the immense potential of this country; the exuberance of Brazilian culture; the generosity of our people; the competence and adherence to ethics of the medical professional and other health professionals in this country; and the fact that renowned professionals in Brazilian institutions gave their seal of approval to this historic event.

There is very strong pressure for similar events to be held again. Those who took part learned about evidence-based medicine, had fun and were able to confirm that there was a complete absence of conflicts of interest. All who participated and collaborated receive our gratitude and recognition of their great contribution towards healthcare quality and ethical improvement of national and international continuing education.

Considering that high-level continuing education without dependence on sponsorship from interested parties is necessary and possible, we hope that our entities can start to hold events that provide continued updating in accordance with maintaining the credibility of our profession. This is essential for all of Brazilian society, including those parts of society that honestly and deservedly depend on profits.

